# Supplementary material for: Association between oral microbiome and seven types of cancers in East Asian population: a two-sample Mendelian randomization analysis
Source: Front Mol Biosci. 2023 Nov 21;10:1327893. doi: 10.3389/fmolb.2023.1327893 (PMC10702768; doi:10.3389/fmolb.2023.1327893)
Supplement: Supplementary file 1 [file DataSheet1.ZIP › Supplementary Materials/MR plots of saliva/saliva═╝/Gastric cancer/pheno.123_to_gastric cancer_forest.pdf]

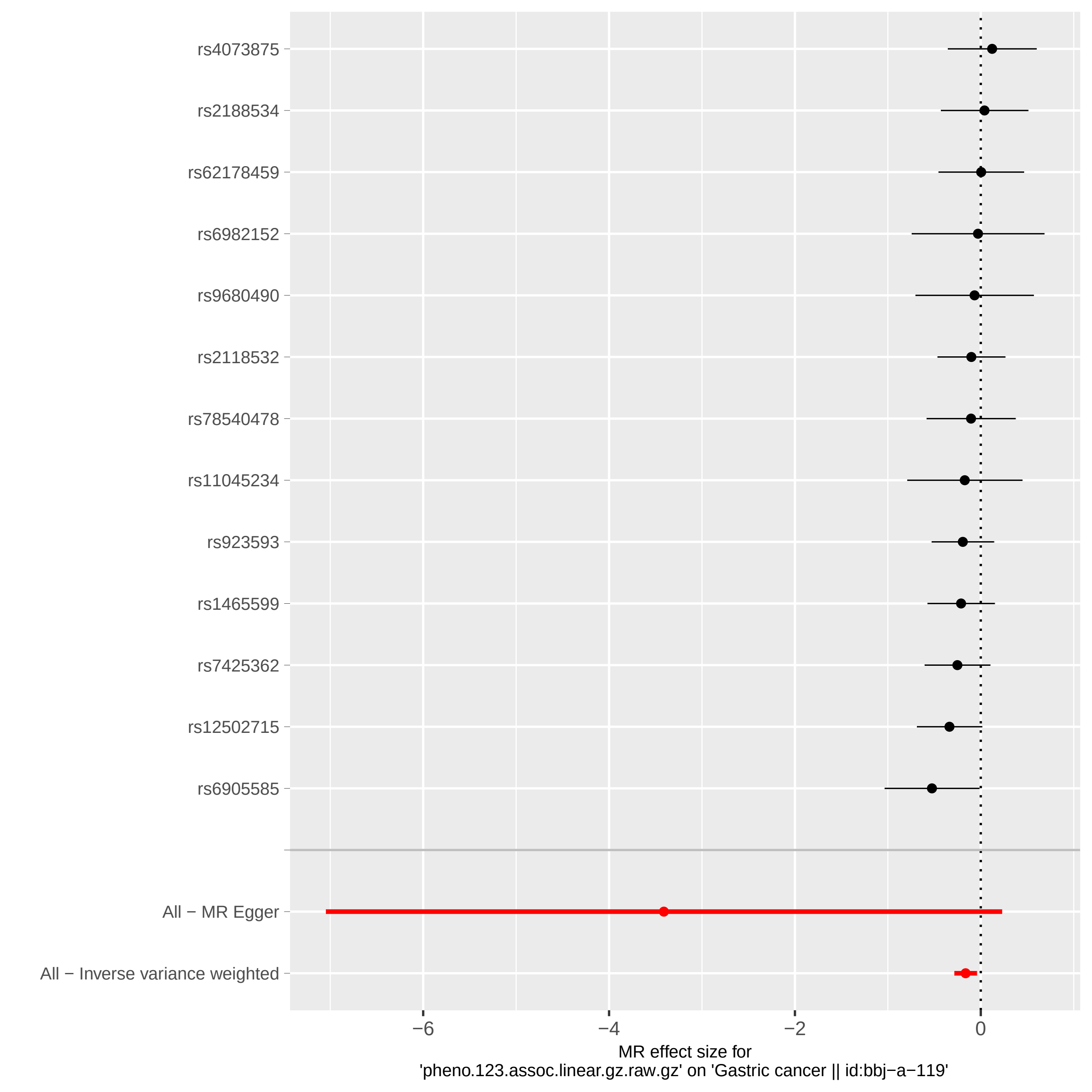
rs4073875
rs2188534
rs62178459
rs6982152
rs9680490
rs2118532
rs78540478
rs11045234
rs923593
rs1465599
rs7425362
rs12502715
rs6905585
All − MR Egger
All − Inverse variance weighted
−6
−4
−2
0
MR effect size for
'pheno.123.assoc.linear.gz.raw.gz' on 'Gastric cancer || id:bbj−a−119'
